# Veterinarians' perspectives of pain, treatment, and diagnostics for bovine respiratory disease in preweaned dairy calves

**DOI:** 10.3389/fpain.2023.1076100

**Published:** 2023-02-23

**Authors:** S. Mijares, L. Edwards-Callaway, I. N. Roman-Muniz, J. F. Coetzee, T. J. Applegate, M. C. Cramer

**Affiliations:** ^1^ Department of Animal Sciences, Colorado State University, Fort Collins, CO, United States; ^2^ Department of Anatomy and Physiology, Kansas State University, Manhattan, KS, United States; ^3^ Department of Clinical Sciences, Colorado State University, Fort Collins, CO, United States

**Keywords:** analgesia, BRD, calf, Identification, pneumonia

## Abstract

**Background:**

Bovine Respiratory Disease (BRD) is a leading cause of morbidity and mortality in preweaned dairy calves. Early detection and therefore treatment are essential to minimize animal welfare concerns, particularly given that recent research also demonstrates that BRD is painful. Veterinarians are essential to ensuring calves with BRD receive appropriate treatment, but little to no research exists regarding veterinarians' perspectives about BRD detection and treatment in dairy calves. This is a critical step to determine education and outreach needs that can target BRD treatment to improve calf welfare. Thus, the objectives of the current study were to describe US veterinarians' current detection methods and treatment practices for BRD in preweaned dairy calves, understand veterinarians' rationale for treatment decisions, and identify gaps in knowledge regarding treatment and management of calf BRD.

**Methods:**

An online survey was sent to two veterinarian-focused list-serves and newsletter. Final responses (*n* = 47) were analyzed using qualitative and quantitative analyses.

**Results:**

On-farm necropsy was the diagnostic tool most considered “extremely important” (26, 55.3%). All veterinarians indicated that BRD was at least mildly painful. However, only 53% of veterinarians (*n* = 25) assess pain in preweaned calves with BRD in order to make treatment decisions. Furthermore, of the veterinarians that assessed pain, 40% (*n* = 10) reported that their knowledge of pain assessment and treatment was adequate, but most (*n* = 24) considered a calf's pain-level at least “moderately important” to make BRD treatment decisions. The most important ancillary therapy for antimicrobials were NSAIDs (21, 44.7%). The ancillary therapy most often considered “extremely important” for treating BRD was NSAIDs. Qualitative analysis identified the following as factors that influenced veterinarians' willingness to provide analgesia: the farm's willingness to administer drugs, clinical signs, perceived severity of pain, the need for anti-inflammatories, and the presence of fever and comorbidities.

**Discussion:**

This study included a small sample size and an extremely low response rate; results should therefore be interpreted with caution. Despite this limitation, important gaps in knowledge were identified, including pain assessment and consideration when making treatment decisions, and diagnostic tools. Addressing these needs in future research and outreach efforts could help ensure appropriate and timely treatment of calf BRD, including pain mitigation.

## Introduction

Bovine Respiratory Disease (BRD) is a term that encompasses both upper respiratory disease and lower respiratory disease (1). While estimates vary, multiple studies have stated that BRD affects approximately 12%–16% of preweaned dairy calves and is responsible for nearly one-fourth of calf deaths (2–4). Negative sequelae of calfhood BRD include, but are not limited to, reduced growth during the preweaned period, decreased milk production during their first lactation, and increased risk for culling prior to completing their first lactation (5–7). Notably, there is mounting evidence that suggests BRD in calves is painful (8, 9). Additionally, calf BRD accounts for a large proportion of total calf antibiotic usage (10). Early and appropriate treatment can help mitigate negative consequences of BRD, including pain, and can improve treatment outcomes (11). However, little is understood about veterinarians' perspectives regarding BRD detection and treatment, which is of interest to determine gaps in knowledge that could be addressed *via* education and outreach which would, in turn, improve calf welfare.

Two tenets of managing BRD include accurate and timely BRD detection as well as appropriate treatment. However, little is known about methods used by veterinarians to detect BRD and if these methods are highly sensitive for BRD identification. For example, auscultation is a skill taught in veterinary education, but has poor sensitivity for BRD identification (12). If BRD goes undetected, calves do not receive appropriate and timely treatment. In addition to prompt detection, an important part of effective BRD treatment includes administration of antimicrobials (11). Over 90% of calves affected with BRD are treated with antimicrobials (4), which therefore contributes to overall antimicrobial usage in cattle (10). Given that the antimicrobials commonly used to treat BRD are under scrutiny due to their role in antimicrobial resistance in human medicine (13, 14), increasing our understanding of how veterinarians use antimicrobials is essential to continually improving judicious antimicrobial usage.

Mitigating pain, including pain due to BRD, is a key component of good animal welfare (15). Pain is an aversive state and a stressor for animals that can cause behavioral changes (16). Veterinarians are ethically responsible for recognizing and responding to pain as part of their veterinary oath to promote animal welfare and relieve animal suffering and this includes mitigating pain during disease (17). Martin et al. (8) demonstrated that calves who developed pneumonia after being inoculated with *Mannheimia haemolytica* showed a combination of decreased activity levels, increased visual analog scale pain scores, and changes to kinematic gait, which indicates bacterial pneumonia in cattle is painful. Blood concentrations of pain biomarkers were greater in calves with induced pneumonia, compared to control calves (9). In addition, there is ample evidence that calves with BRD show behavioral changes such as more time lying down and reduced milk intake, as well as reduced growth (7, 9). Thus, understanding how veterinarians are addressing pain associated with BRD is key to addressing potential animal welfare concerns.

One class of drugs that veterinarians have at their disposal to mitigate pain are non-steroidal anti-inflammatory drugs. The drug flunixin meglumine is the only NSAID approved for use to control fever from respiratory disease in cattle in the United States (18). NSAIDs have been successful at relieving pain associated with painful procedures such as castration and disbudding (19). Despite evidence that BRD is painful, little to no data are available to understand effective pain mitigation strategies for BRD. A literature review from Francoz et al. in 2012 concerning the use of NSAIDs as ancillary therapy for BRD found that NSAIDs resulted in faster temperature drops, reduced lung consolidation, decreased respiratory rate, and increased weight gain compared to antimicrobial treatment alone in some studies. Improved performance (e.g., including increased weight gain) after NSAID administration might indicate reduced pain and thus increased animal welfare, though data is currently limited (20).

Data regarding veterinarians' perspectives pertaining to BRD detection and treatment are lacking. However, given the pervasiveness of BRD in dairy calves and growing evidence that BRD is painful, understanding veterinarian's perspectives is key to identifying critical gaps in knowledge. Therefore, the objectives of the current study were to describe veterinarians' current detection methods and treatment practices for BRD in preweaned dairy calves, understand veterinarians' rationale for treatment decisions, and identify gaps in knowledge regarding treatment and management of calf BRD.

## Materials and methods

The study materials and research plan were approved through the Colorado State University (CSU) Institutional Review Board (#1826) prior to project initiation.

### Survey development and content

A survey regarding veterinarian perspectives of the detection, treatment, and pain associated with Bovine Respiratory Disease (BRD) was developed by the authors, who have expertise in calf health, animal welfare, clinical pharmacology, veterinary medicine, and survey question development. The survey was developed in Qualtrics survey software (Qualtrics, Provo, UT, United States) and pretested by two veterinarians (expert reviewers who represented our target population) and four graduate students (peer reviewers familiar with the survey topic) who were not involved with the initial drafting of questions. Pre-testers assessed questions for clarity, relevance to study objectives, and content as described by Rattray and Jones (21) and as done in previous studies (e.g., 22, 23). Feedback from the pre-testers was used to refine the survey format and questions. The survey sent to pre-testers was 37 questions; the final survey consisted of 35 questions though a variable number of questions were displayed for participants based on branch and display logic. The anticipated completion time was 15–20 min. The survey contained a variety of question types including multiple choice, open-ended, ranking, and select all that apply. The survey included blocks with questions regarding demographics, pain perspectives related to BRD and fever, and BRD detection, treatment, and control (see Supplementary Materials). To ensure consistent definitions of BRD, the following statement was included at the beginning of the survey: “In this survey, BRD is used to mean upper and/or lower respiratory tract disease, including pneumonia, in preweaned dairy calves”. For the question: “How important (from not at all important to extremely important) are each of the following when assessing pain associated with BRD in preweaned dairy calves?”, respondents received a list of previously described signs of pain (24, 25) that were adapted for this survey; respondents were asked to indicate their perceived level of importance for each sign of pain. “Fever” was not defined in the survey and was therefore open to interpretation by respondents.

### Study population and survey dissemination

The population of interest for this study was practicing veterinarians in the United States who regularly worked with preweaned dairy calves. In order to target this population, the first distribution of the survey was sent to all AABP members of the American Association of Bovine Practitioners (AABP) in their newsletter (*n* = 4,000) and additionally to those members who are also on the email listserv (*n* = 1,757) in April 2021. The second distribution was sent to 3,364 people *via* a newsletter distribution list for *Bovine Veterinarian* magazine in June 2021; this distribution list contained veterinarians as well as non-veterianrians*.* Both distributions remained open for 30 days each, and one reminder email was sent at approximately 14 days after the initial message. All invitations to participate were sent as emails with a direct survey link. No incentive to participate was provided. No identifying information was collected with any responses. All questions were optional and could be skipped by the respondent, except the first question to obtain consent to participate in research. Contact information for the PI of this study was provided if respondents had any questions. A question regarding the US region in which the participant practiced was included.

### Statistical analysis

Survey data was exported from Qualtrics to Microsoft Excel (Microsoft Corporation, Redmond, WA). Any surveys <80% complete were excluded from analysis. Due to the small sample size of this survey, only descriptive summary statistics were performed. Results are reported as (*n*, percentage) unless otherwise noted.

### Thematic analysis

To fully explore open-ended responses in this survey, thematic analysis methods described by Braun and Clarke (26) were used. Three researchers reviewed all open response questions and identified initial themes and theme definitions. The same team of researchers then independently coded all responses for identified themes. Coding validation was achieved by comparing results and discussing any disagreement until a common set of themes was agreed upon for each response.

## Results

This survey was distributed to 9,121 names on listservs and received 55 responses for an estimated response rate of 0.6%; it is likely there was overlap of people between distribution lists. Any responses that were less than 80% complete were excluded from analysis, thus a total of 47 responses were included in this data set. All (47, 100%) respondents of this survey were veterinarians practicing in the US. Twenty-eight percent of respondents were 25 to 34 years old (*n* = 13), 23% were 35 to 44 years old (*n* = 11), 13% were 45–54 (*n* = 6), 19% were 55–64 (*n* = 9), and 17% were 65 years or older (*n* = 8). A small majority of respondents were female (24, 51.0%). The largest group of survey respondents had practiced veterinary medicine for more than 20 years (22, 46.8%) and were based in the Midwest region of the United States (27, 57.4%).

### Quantitative analysis

**Figure 1:**
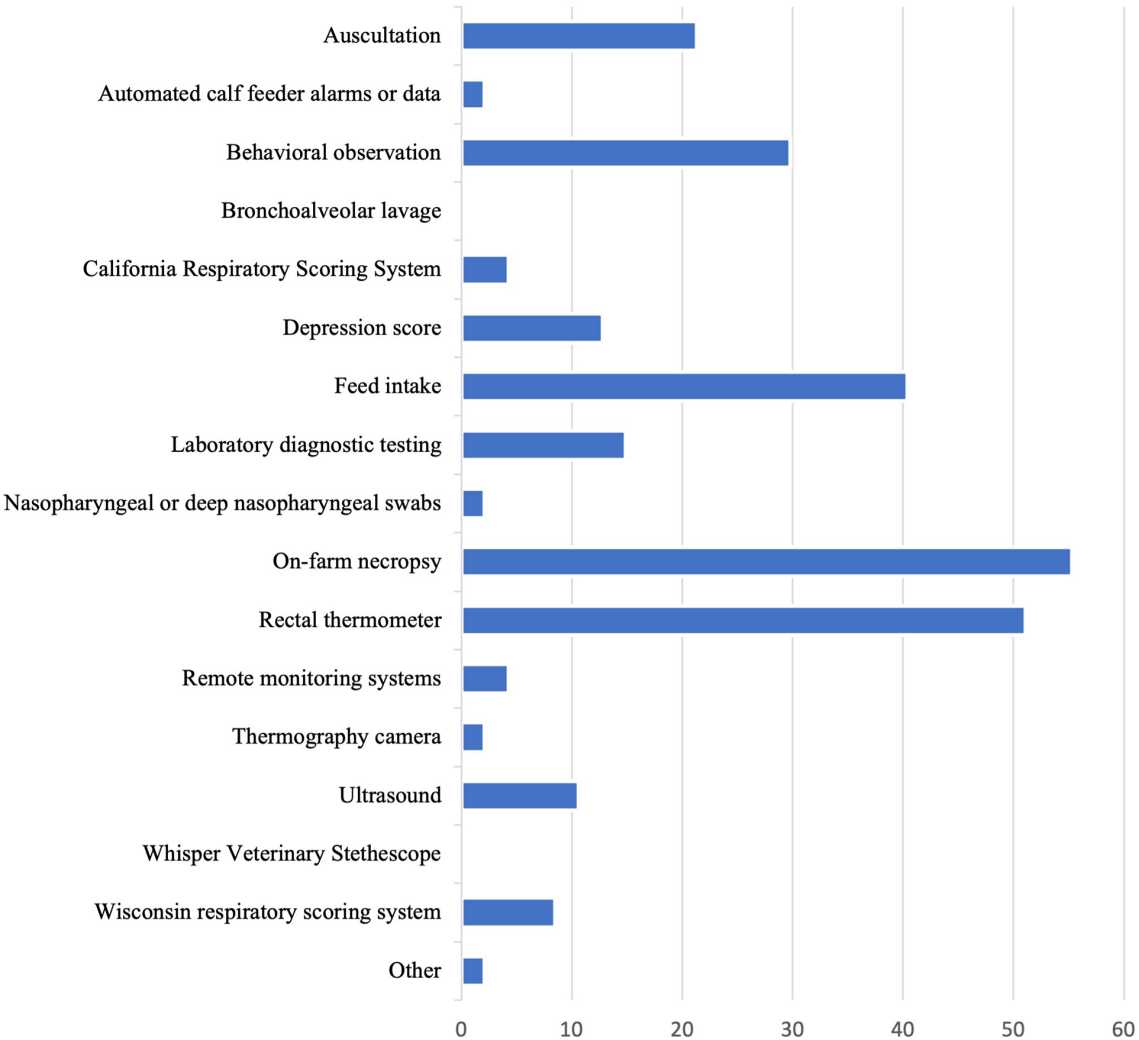
Percentage of veterinarians that consider each of the following diagnostic tools “extremely important” in reaching a diagnosis of BRD in preweaned calves.

**Figure 2:**
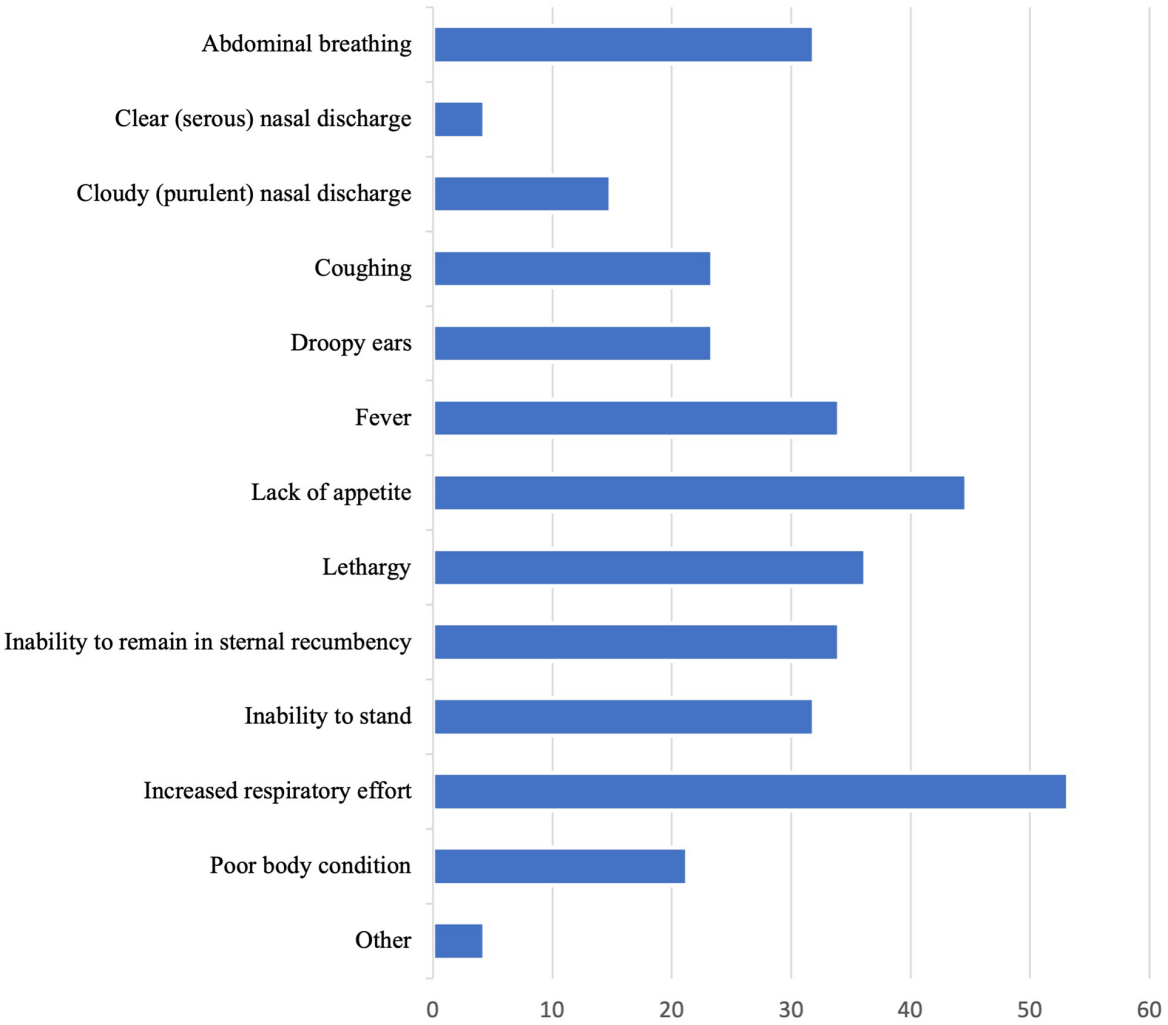
Percent of veterinarians that find each of the following clinical signs "extremely important" in reaching a diagnosis of BRD in preweaned calves.

The diagnostic tool most commonly considered “extremely important” in making a diagnosis of BRD was on-farm necropsy of deceased animals (26, 55.3%) (Figure 1). The tool most considered “not at all important” was the Whisper*®* Veterinary Stethoscope (32, 68.1%). Increased respiratory effort was the clinical sign most considered “extremely important” in making a diagnosis of BRD (25, 53.2%; Figure 2). The clinical signs most considered “not at all important” were clear or serous nasal discharge (3, 6.4%) and poor body condition (3, 6.4%).

**Figure 3:**
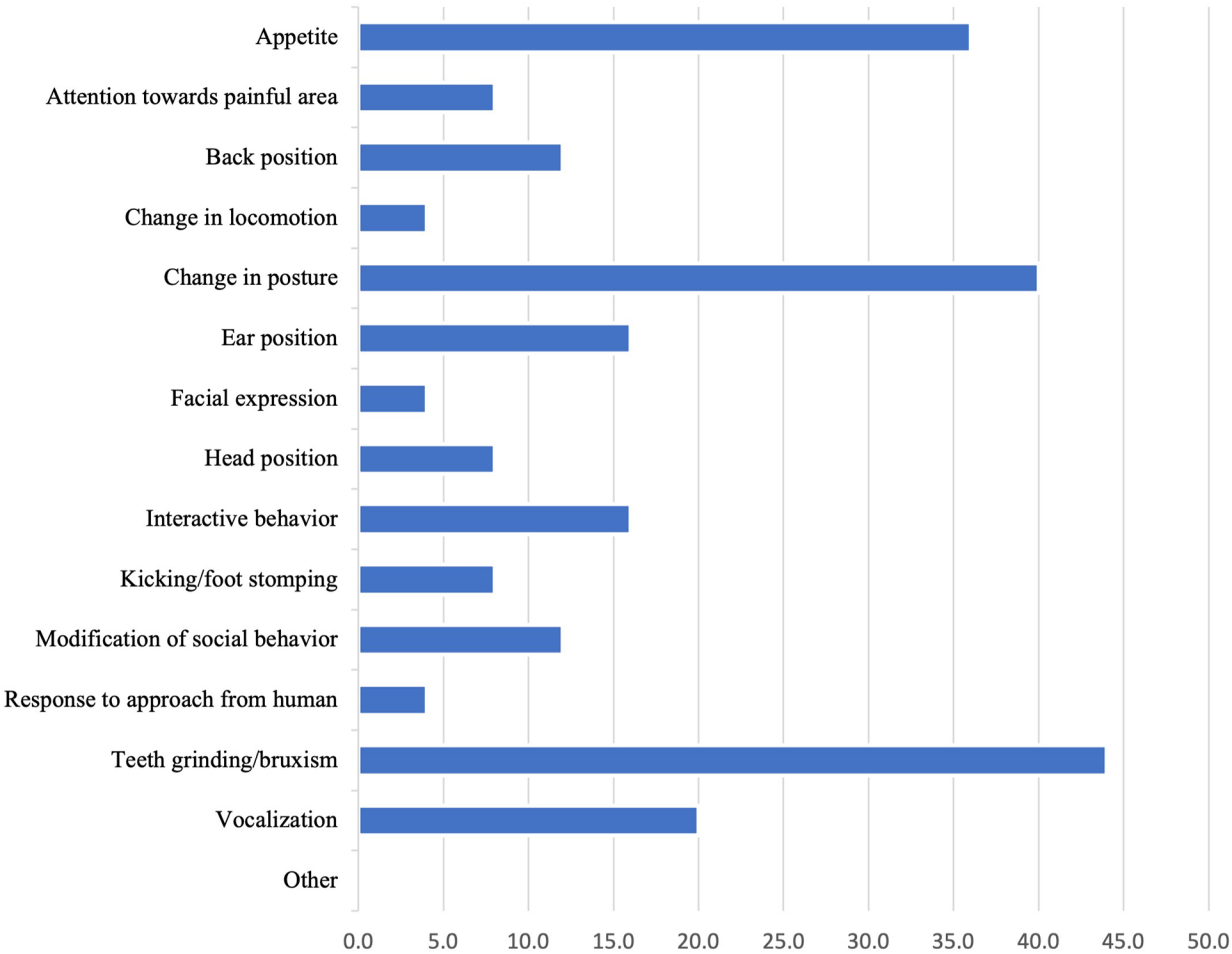
Percentage of veterinarians that consider each of the following “extremely important” for assessing pain in preweaned calves with BRD. This question was available to respondents who indicated they assessed pain to make treatment decisions (*n* = 25).

For the question, “How painful do you consider BRD (pneumonia) to be for preweaned calves?”, all respondents indicated that there was at least mild pain associated with BRD, with 66% (*n* = 31) responding that BRD was moderately painful (Table 1). Most veterinarians also consider fever alone to be moderately painful (26, 55.3%). Just over half of veterinarians (25, 53.2%) assess pain in preweaned calves with BRD in order to make treatment decisions. Of the veterinarians who did assess pain, the factor most commonly considered “extremely important” for assessing pain was teeth grinding or bruxism (11, 44.0%) (Figure 3). Although bruxism was considered extremely important for assessing pain, a small proportion (2, 8.0%) identified this as a least important factor. Of the veterinarians that assess pain to make BRD treatment decisions, 40% (*n* = 10) considered their knowledge of recognizing and treating pain in preweaned dairy calves to be adequate. The majority of respondents who assessed pain also considered the animal's current pain-level to be at least “moderately important” when making treatment decisions regarding BRD for preweaned calves (Table 2).

**Table 1:** Percentage of respondents in each category for the question “How painful do you consider BRD (pneumonia) to be for preweaned calves?”.

Perceived pain level	% (*n*)
No pain	0
Mild	13 (6)
Moderate	66 (31)
Severe	19 (9)
Very Severe	2 (1)
Worst pain imaginable	0

**Table 2:** Percentage of respondents in each category for the question “how important is the animal's current pain-level when making a treatment decision regarding BRD for preweaned calves?”. This question was available to the 25 respondents that indicated they assessed pain in preweaned calves with BRD in order to make treatment decisions.

Perceived importance	% (*n*)
Not at all important	4 (1)
Slightly important	0
Moderately important	28 (7)
Very important	44 (11)
Extremely important	20 (5)

The majority of veterinarians (25, 55.3%) administer 2 antimicrobial treatments to a typical case of BRD in preweaned dairy calves before clinical signs resolved. The largest percent of veterinarians (17, 36.2%) typically wait 4 to 7 days before determining that the previous antimicrobial treatment was unsuccessful in the presence of continuing clinical signs. The most common antimicrobial chosen for first-line treatment for BRD was Tulathromycin (e.g., Draxxin®100mg; 19, 40.4%) while Florfenicol (15, 31.9%) was the most common selection for a second-line treatment. The most common reasons for using the first-line treatment were antimicrobial sensitivity of farm-specific pathogens (14, 29.8%) and personal experience demonstrating effectiveness at resolving BRD (14, 29.8%). The majority of veterinarians (33, 70.2%) indicated that they did not use antimicrobials in an extralabel manner to treat BRD.

**Figure 4:**
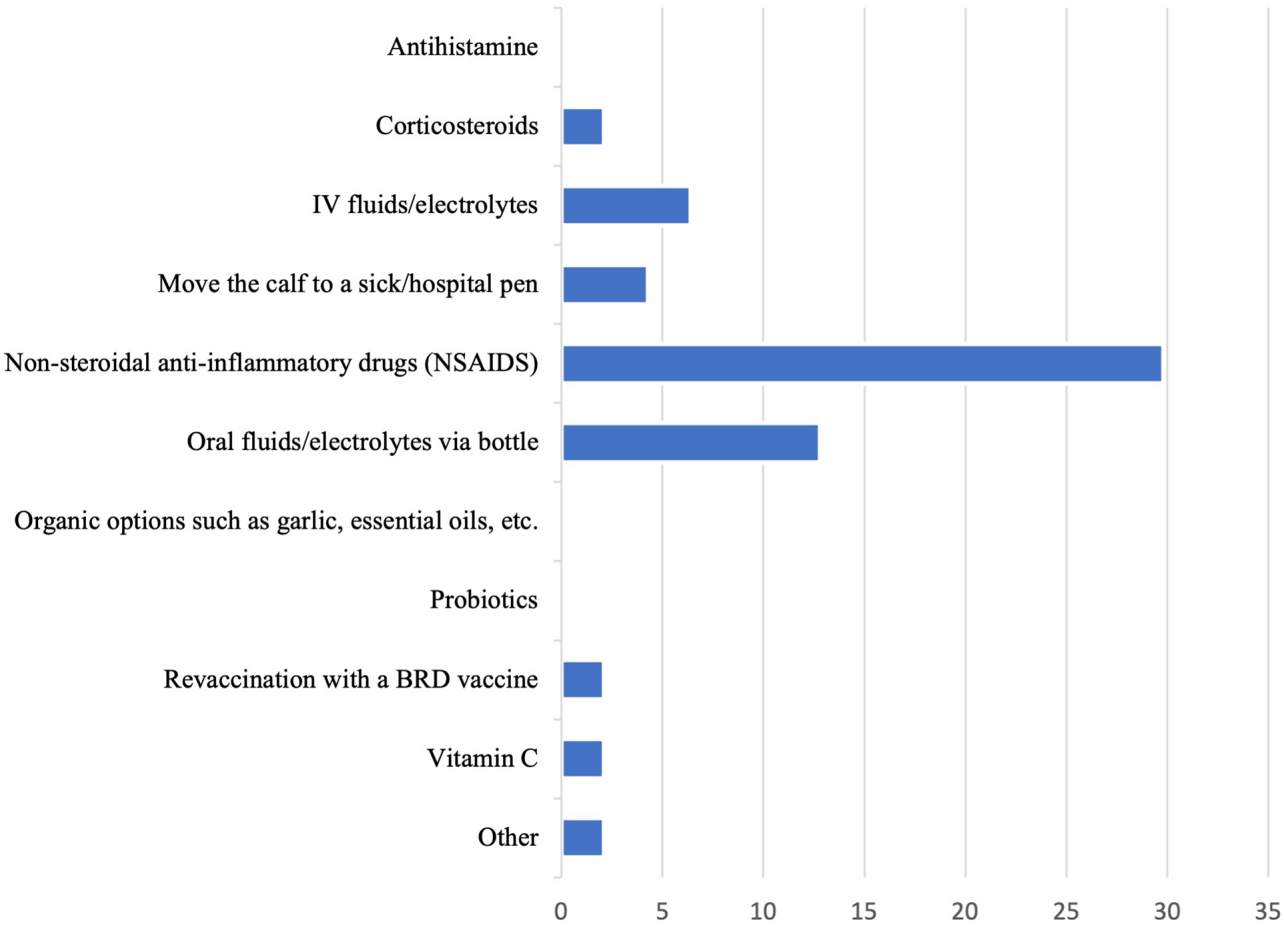
Percentage of veterinarians that consider the following ancillary therapies, in addition to antibiotics, “extremely important” in the treatment of BRD for preweaned calves.

Given the choice between an antimicrobial alone, NSAID alone, or combination antimicrobial with NSAID, most veterinarians indicated they would choose a combination antimicrobial with NSAID (28, 59.6%). When asked “How frequently do you use drugs (other than antimicrobials) to reduce fever in a preweaned calf with BRD?”, 15% (*n* = 7) selected “always”, 43% (*n* = 20) selected “most of the time”, 28% (*n* = 13) selected “about half the time”, 13% (*n* = 6) selected “sometimes”, and no respondents selected “never”. Besides antimicrobials, the most important ancillary therapy that veterinarians included in their treatment regimen for BRD were NSAIDs (12, 26%; Figure 4). The ancillary therapy most considered “not important at all” was organic options such as garlic or essential oils (40, 85.1%). The most important reason cited for choosing to administer ancillary therapy to a preweaned calf with BRD was efficacy at resolving BRD (16, 34.0%), while the least important reason was cost (9, 19.1%), though about one third of veterinarians identified that cost is moderately important (16, 34.0%). For the question “please rank your top 3 non-steroidal anti-inflammatory drugs that you, as a veterinarian, would select when treating BRD in preweaned dairy calves that meet your case definition”, 68% of respondents (*n* = 32) indicated that flunixin injectable was their 1st choice, 36% indicated oral meloxicam was their 2nd choice (*n* = 17), and 17% (*n* = 8) indicated that flunixin pour-on was their third choice.

### Qualitative analysis

Themes and responses of questions selected for thematic analysis are described below in Tables 3–5. When asked to explain how painful they considered BRD to be for preweaned dairy calves, 46.5% of respondents used clinical signs as a descriptor (Table 3). The theme of “Clinical signs” often appeared in tandem or linked with the theme of “Ungraded pain and discomfort” (Table 3). For the question “Please explain your answer regarding how painful you consider fever to be for preweaned dairy calves”, many people who considered fever to be painful anthropomorphized how the calf was feeling based on their own experiences of having a fever previously (Table 4). For the question “How do you decide if analgesics are warranted for a preweaned calf with BRD?”, many responses linked the categories of “sometimes” using analgesics with what clinical signs the calves were demonstrating (Table 5). For the question “What do you think future studies regarding calf health need to address?”, respondents indicated studies regarding the immune response of calves, effects of treatment decisions on calf health, use of ultrasound, vaccines, NSAID, and studies on management factors including stocking density and pen size as important future directions.

**Table 3:** Summary of themes and percentage of responses for the question “please explain your answer regarding how painful you consider BRD to be for preweaned dairy calves (*n* = 34 responses).

Theme	Definition	Percentage of Responses	Quote
Clinical signs	Any observed symptom exhibited by calf that demonstrates the calf may be in pain.	46.8%	“*Difficulty breathing and increased respiratory rates”*
Environment	Surroundings or conditions around the calf.	2.1%	*“The causative agent, comorbidities, and the environment all play a role.”*
Duration or severity	The time period of the disease or extent to which the animal is sick with BRD.	23.4%	*“The level of pain is most likely correlated to how severely affected they are with BRD.”*
Fever	Increased body temperature.	8.5%	“*High fever >104.5°F.”*
Comparison to human experience	Direct comparison to own experience or other anthropomorphization.	14.9%	*“I have had pneumonia and it is very painful.”*
Mild pain	Specific mention of being mildly painful.	8.5%	*“A mild case caught early would not be that painful.”*
Moderate pain	Specific mention of being moderately painful.	6.4%	*“There is inflammation which I believe is moderately painful.”*
Severe pain	Specific mention of being severely painful.	12.8%	*“A chronic case caught late could be very painful.”*
Ungraded pain, uncomfortable, discomfort	Mention of pain that is not specifically mild, moderate, or severe; mention of discomfort.	36.2%	*“Not so much painful as uncomfortable.”*

**Table 4:** Summary of themes and percentage of responses for the question “please explain your answer regarding how painful you consider fever to be for preweaned dairy calves (*n* = 33 responses).

Theme	Definition	Percentage of Responses	Quote
Clinical signs	Any observed symptom exhibited by calf that demonstrates the calf may be in pain.	21.3%	*“Fever certainly causes depression and depression of feed intake…”*
Duration or severity	The time period of the disease or extent to which the animal is sick with BRD.	19.1%	*“It will depend on the severity of the fever.”*
Comparison to human experience	Direct comparison to own experience or other anthropomorphizing.	12.8%	*“I feel moderately painful when I have a fever, and thus I pass that feeling on to calves with fevers.”*
Mild pain	Specific mention of being mildly painful.	0%	-
Moderate pain	Specific mention of being moderately painful.	2.1%	*“I feel moderately painful when I have a fever.”*
Severe pain	Specific mention of being severely painful.	2.1%	*“High fever would be very painful, as it is in people.”*
No pain	Specific mention of BRD not being painful.	8.5%	*“I believe that fevers itself are not painful.”*
Ungraded pain, uncomfortable, discomfort	Mention of pain that is not specifically mild, moderate, or severe; or mention of discomfort.	31.9%	*“Its uncomfortable but not a true pain experience.”*

**Table 5:** Summary of themes and percentage of responses for the question “how do you decide if analgesics are warranted for a preweaned calf with BRD? (*n* = 40 responses).

Theme	Definition	Percentage of Responses	Quote
Always with explanation	Respondent indicated that they always gave analgesics and may have provided an explanation why.	14.9%	*“I personally advocate client's to always use an anti-inflammatory.”*
Sometimes, it depends with explanation	Respondent indicated that they administered analgesics sometimes and then provided an explanation.	63.8%	*“I decide based on the severity of the fever and clinical signs as well as comorbidities.”*
Farm's willingness	Explanation mentioned analgesic administration was dependent upon the farm's willingness to administer or pay for analgesia.	6.4%	*“Unfortunately, it's farm specific if they are willing to take the time to administer.”*
Clinical signs	Analgesic administration was dependent upon observed symptom exhibited by calf that demonstrates the calf may be in pain.	31.9%	*“presence and level of fever, indications of pain from calf.. anorectic, lethargic, grunting…”*
Pain, discomfort, uncomfortable	Analgesic administration was dependent upon pain of any type or severity, discomfort, or calf being uncomfortable.	12.8%	*“Only if the fever cannot be controlled using antibiotics alone or if there is discomfort for the calf.”*
Anti-inflammatory	Analgesic medication was also used for anti-inflammatory effects.	4.3%	*“I tend to use banamine as an antitoxin and anti-inflammatory – which I guess would reduce pain.”*
Fever	Analgesic administration was dependent upon increased body temperature.	38.3%	*“If a fever is present, if calf is coughing, depressed and demonstrating labored breathing I advocate for NSAID use based on fever reducing properties and pain relief from respiratory disease.”*
Severity, co-morbidities	Analgesic administration was dependent upon severity, other diseases, or co-morbidities.	19.1%	*“Degree of fever and relative risk for GI ulceration (other comorbidities).”*
Never and explanation	Respondent indicated that they never gave analgesics and may have provided an explanation why.	2.1%	*“I usually don't select an analgesic drug for BRD treatment.”*

## Discussion

Literature describing veterinarians' current detection and treatment methods for BRD in preweaned dairy calves is limited, and literature regarding veterinarians' perspectives of pain associated with BRD in preweaned calves is especially lacking. Previous studies have focused on the development of novel risk assessment tools for BRD (27), assessing quality of life for calves with BRD (28), and validating diagnostic tools (29) but research focused on veterinarians' perspectives of the disease and pain associated with the disease is limited. The intent of this study was to describe veterinarians' current detection methods for BRD in preweaned dairy calves, describe veterinarians' current treatment practices for BRD in preweaned dairy calves, understand veterinarians' rationale for treatment decisions and identify gaps in knowledge regarding treatment and management of calf BRD.

In this study, the largest proportion of respondents (27.7%) were 25–34 years old while the next largest proportion (23.4%) were 35–44 years old. This compares favorably to the American Veterinary Medical Association (AVMA) census of veterinarians, which finds that 39% of veterinarians are currently between the ages of 25 and 40 (30). A small majority of survey respondents were female (51.0%). Though the 2018 AVMA census of veterinarians found 61.7% of veterinarians were female, the field of food animal medicine is male predominant as 77.1% of veterinarians in food animal exclusive veterinary practices and 74.3% of veterinarians in food animal predominant veterinary practices are male (30).

This study aimed to identify information specific to preweaned calves in a way that was reflective of day-to-day realities on farms. Summary statistics from this survey are valuable as limited literature exists describing veterinarians’ detection and treatment for BRD in preweaned dairy calves, or veterinarians' perspectives of pain associated with BRD. Due to the small sample size of this survey, limited conclusions can be drawn, and results should be interpreted with appropriate caution. This survey was specific to veterinarians who currently worked with and made treatment decisions for preweaned dairy calves, thus the small sample size of this survey was likely due, in part, to the relatively small target population. For example, of the current members in the American Veterinary Medical Association (AVMA), only 5.6% work in either food animal exclusive or food animal predominant clinical practices (30). Although we did not directly survey members of AVMA *via* a member listserv, AVMA provides information regarding US veterinarian demographics, which to our knowledge is not available for other organizations (e.g., AABP). Survey participation was also limited to veterinarians in the US, which allowed for an improved understanding of perspectives of pain, treatment, and diagnostics of BRD in calves on US dairies. Given that countries outside the US have different regulations surrounding pain mitigation in livestock, limiting responses to veterinarians that practice in the US provides information most germane to the US dairy industry. In addition, the survey was only disseminated online to AABP members and the Bovine Veterinarian Magazine members, which meant that veterinarians who were not on these lists did not receive the survey, nor did those in areas with poor internet access. Furthermore, incentives for participation were not provided which could have increased the response rate. Finally, this survey was disseminated during the COVID-19 pandemic, which could have limited veterinarians' willingness or ability to participate. The low response rate among veterinarians in the present study is similar to the response rate in other studies that disseminated surveys to people in the agriculture industry during the pandemic (e.g., 23, 31, 32). This may be due to the additional demands and stressors veterinarians and agriculture workers experienced during the COVID-10 pandemic (33–35). Future studies should explore a variety of methods (e.g., mail surveys, incentives, and in-person recruitment) and consider optimal timing of dissemination to increase the survey response rate.

### Diagnostic tools

Accurate diagnostic tools are key to identifying calves with BRD so that they can receive prompt treatment. The diagnostic tool most considered “extremely important” by veterinarians in this study for making a BRD diagnosis was on-farm necropsy of deceased animals (55.3%) By relying on post-mortem diagnostics, veterinarians may miss opportunities for early identification and intervention of BRD. We recognize that the phrasing of the question did not allow for us to identify the most common pre-mortem BRD identification methods. However, the 2nd and 3rd diagnostic tools most often ranked as "extremely important" in reaching a diagnosis of BRD in preweaned calves were rectal thermometer and feed intake, respectively. Although fever is common in calves with clinical BRD, a previous study reported that about two-thirds of calves with subclinical BRD (no clinical signs, but ≥1 cm^2^ of lung consolidation identified on lung ultrasound) did not have a fever (7). Additionally, feed intake as an indicator of illness may only be reliable when calves are on a high plane of nutrition, as calves on a low plane of nutrition do not typically decrease milk intake when ill (36). Methods that could improve sensitivity of BRD detection pre-mortem, such as laboratory diagnostic testing, bronchoalveolar lavage, and nasopharyngeal or deep nasopharyngeal swabs were not considered extremely important diagnostics by the majority of respondents, perhaps because these methods are more complicated and expensive to perform on-farm. Lung ultrasound, which has been previously validated, is practical for calf-side use, and has a high sensitivity and specificity for detecting pneumonia in preweaned dairy calves (37), was considered “extremely important” by only 10% of veterinarians in the study. Thus, an additional gap in knowledge is identified: increasing veterinarians' knowledge regarding accurate pre-mortem diagnostic tools could improve the industry's ability to identify BRD early and therefore provide appropriate and timely treatment.

### Pain assessment

Assessing pain is an important step to ensure livestock receive appropriate treatment, as it is the initial assessment that can influence treatment decisions. Most veterinarians in the present study reported that BRD was at least mildly painful, with 66% responding that BRD was moderately painful. However, only 53% of veterinarians surveyed assessed pain in preweaned calves with BRD and only 40% of that subset (*n* = 10) considered their ability to assess pain “adequate”. The disconnect between perceived level of pain and pain assessment (including the ability to assess pain) signifies an important opportunity to increase veterinarians' skills for pain assessment through further training and education. Robles et al. (38) conducted a survey of veterinarians that treat dairy and/or beef cattle in the US and 69% of veterinarians in that survey considered themselves “knowledgeable about recognizing and treating pain in cattle”. The difference between the present study and Robles et al. (38) could be due to the difference in how the survey questions were written, i.e, Robles et al. (38) asked if respondents were knowledgeable, whereas the present study asked if they considered their ability to assess pain as “adequate”. However, it could also be due to the population of animals- Robles et al. (38) was for dairy and/or beef cattle of all ages and the present study was specific to preweaned calves. Regardless, both studies demonstrate that there is a proportion of veterinarians who do not consider themselves knowledgeable, or that their ability is adequate, when assessing pain in cattle.

Assessing pain in livestock species is notoriously challenging due to their stoicism as prey species and their proclivity to mask signs of pain (39). Furthermore, pain assessment methods such as blood biomarkers, gait velocity measured using pressure mats, activity and rumination levels, infrared thermography (8, 40), although useful to assess pain in research setting, may not be practical to assess pain calf-side when diagnosing BRD on-farm. Millman (41) suggests that when behaviors are clearly explained and validated, they can be a robust method to assess pain. However, validated and clearly explained behavioral measures of pain, that are also practical for on-farm use, are lacking (41). For example, in the present study, the two factors that the largest percentage of respondents considered “extremely important” for assessing pain associated with BRD are subjective and could vary depending on observer (teeth grinding or bruxism: 44%; change in calf posture: 40%). The objectivity and validity of behavioral observations to assess pain can be improved when training materials (e.g., definitions, pictures, and videos) are provided to observers (41). As such, future research should identify pain assessment methods that are practical for use on-farm and in which behaviors are well-defined. Pain assessment methods should also include training materials to ensure that observers are able to correctly assess pain. Importantly, pain assessment methods for BRD should be integrated into veterinary education to ensure practitioners have the necessary skills to assess pain and provide appropriate treatment.

### Pain relief

Mitigating pain is a key component of veterinary medicine and is included in the veterinary oath (15, 17). Although all veterinarians from the current survey indicated that BRD was at least mildly painful and that 64% considered the animal's current pain level as either “very important” or “extremely important”, only about 30% of respondents indicated that providing NSAIDs was “extremely important” in the treatment of BRD and analgesia was not always administered. In a previous survey of beef and dairy producers, producer-veterinarians, and veterinarians, most respondents reported that BRD in cattle less than 2 months of age was at least moderately painful and the likelihood of providing pain mitigation increased with perceived pain severity (Edwards-Callaway et al., in press). Furthermore, respondents’ perceptions of pain level were influenced by gender and role in the industry whereby men and producers were less likely to perceive BRD as painful compared to women and producer-veterinarian or veterinarian (Edwards-Callaway et al.; *under review*). Results from the qualitative analysis in the present study provide further insight about why pain relief is not more commonly used; most respondents indicated that their willingness to provide analgesia was dependent upon numerous factors, including the farm's willingness to administer analgesics, clinical signs, perceived severity of pain or discomfort, the need for anti-inflammatory medications for a particular case, if fever is present, and comorbidities. Data demonstrating the benefits of providing analgesia during BRD, particularly situations in which analgesia would be most beneficial (e.g., fever, comorbidities) could potentially make veterinarians and their clients more willing to provide pain mitigation. For example, a previous literature review identified some studies that showed NSAID administration in conjunction with antimicrobials as ancillary treatment for BRD results in faster temperature drops, reduced lung consolidation, decreased respiratory rate, or increased weight gain; however, findings are inconsistent (20). Additionally, increasing our understanding of the barriers to providing pain relief (e.g., cost, extra-label drug usage, and administration logistics; 38) is essential to widespread adoption in the industry. An additional avenue to increase the provision of pain relief would be to target veterinarians’ skills and perceptions; for example, previous work found that the likelihood of providing pain relief to cattle for a variety of conditions increased with increased perception of pain (Edwards-Callaway et al., unpublished data). Few veterinarians in the present study felt their ability to accurately assess pain was adequate. Thus, if veterinarians are more skilled at assessing pain in calves with BRD, they might be more likely to perceive the animal is in pain, and therefore could be more likely to provide analgesia. Efforts to improve veterinarians’ skill sets in assessing pain could be targeted at both veterinary schools and continuing education courses. Doing so would also mean that veterinarians would be more apt to communicate with or train other animal caretakers on pain assessment protocols, as these caretakers could assess calves on a daily basis.

### Antimicrobial use

Bovine respiratory disease is a leading cause of death in preweaned dairy calves and producer-reported data estimates that 24% of dairy calf mortality is due to BRD and approximately 95% of calves identified with BRD are treated with antimicrobials (4). The majority of veterinarians (55.3%) in the present study administered 2 antimicrobial treatments to a typical case of BRD before clinical signs resolved. Multiple treatments for a case of BRD in calves is not unusual (10, 42–44); for example, approximately 24% of preweaned heifers in one study required re-treatment (44). However, re-treatments are not ideal because they contribute to the overall amount of antimicrobials used in dairy calves (10). Judicious antimicrobial usage is increasingly important, particularly because the antimicrobial classes used to treat about half of preweaned calves with BRD are the same classes that are known to increase antimicrobial resistance in humans (4, 10, 45). Ollivett (10) suggests that current antimicrobials administered on-label do not fully resolve bacterial infections in the lung. Incomplete resolution at the lung level is partly due to drug manufacturers using treatment success definitions that are based solely on clinical signs and rectal temperature, which have been shown to misclassify calves with pneumonia (10, 46, 47). In order to provide effective treatment and improve judicious antimicrobial usage, future research efforts should focus on expanding efficacy definitions in drug trials to include direct measures of lung health. Additionally, veterinarians are at the forefront of responsible antimicrobial usage; exploring the challenges veterinarians face regarding treatment failure would also be useful to improve treatment efficacy and reduce antimicrobial usage.

## Conclusion

This is the first study to report veterinarians' perspectives of diagnostics and treatment for bovine respiratory disease in dairy calves. Although there were a small number of respondents and results should be interpreted cautiously, we identified critical disconnects that future research and outreach efforts should address. First, although most respondents reported that BRD was at least mildly painful, only a small proportion of veterinarians in the survey assessed pain in calves with BRD. Second, only a portion of veterinarians considered the animal's pain level when treating, despite most reporting there was some level of pain that accompanies BRD. These disconnects should be addressed in future research efforts that: (1) describe how veterinarians assess pain associated with BRD, (2) identify practical, accurate, and well-defined pain assessment tools for BRD, and (3) identify barriers to providing pain mitigation in calves with BRD. Additionally, there is an opportunity to increase veterinarians' knowledge about accurate and sensitive pre-mortem BRD diagnostic tools and improve their skillset for assessing antimicrobial treatment efficacy. Improving veterinarians' abilities to assess pain and accurately detect BRD could help increase their awareness of pain associated with BRD, and thus increase the likelihood of providing pain relief to calves with BRD.

## Data Availability

The raw data supporting the conclusions of this article will be made available by the authors, without undue reservation.
