# Supplementary material for: Veterinarians' perspectives of pain, treatment, and diagnostics for bovine respiratory disease in preweaned dairy calves
Source: Front Pain Res (Lausanne). 2023 Feb 23;4:1076100. doi: 10.3389/fpain.2023.1076100 (PMC9997724; doi:10.3389/fpain.2023.1076100)
Supplement: Supplementary file 1 [file Datasheet1.docx]

Veterinarian_NSAID_Survey

Start of Block: Introduction

Colorado State University is conducting a research survey of practicing dairy veterinarians to identify current detection and treatment strategies for preweaned dairy calves with bovine respiratory disease (BRD).    Thank you for taking the time to fill out this research survey. For each question that has a fill-in option, you are welcome to add your own selection if you feel it is not best described by one of the choices. The open-ended questions are intended for you to share your personal opinions or experiences. We anticipate the survey should take 15-20 minutes to complete.    Your feedback is anonymous and will not be used for any purpose other than for our research survey questions. There are minimal risks to this research survey and you may choose to stop at any time.**In this research survey, BRD is used to mean upper and/or lower respiratory tract disease, including pneumonia, in preweaned dairy calves.**    The goal of this research survey is to understand current BRD treatment and detection strategies of in preweaned calves. Specifically, the objectives of this research survey are:  1.        describe veterinarians’ current treatment practices for BRD in preweaned dairy calves  2.        describe veterinarians’ current detection methods for BRD in preweaned dairy calves 3.        understand veterinarians’ rationale for treatment decisions  4.        identify gaps in knowledge regarding treatment and management of calf BRD    Thank you for taking this survey. If you have any questions about this research project, please contact:   Dr. Catie Cramer, MS, PhD (Principal Investigator) Assistant Professor Department of Animal Sciences Colorado State University catie.cramer@colostate.edu.   Dr. Tanya Applegate, MS, DVM, DACVIM (LAIM) Instructor Equine Medicine and Livestock Veterinary Services Colorado State University   Dr. Hans Coetzee, BVSc, Cert CHP, PhD, DACVCP, DACAW, DECAWBM Professor and Head Department of Anatomy & Physiology Kansas State University   Dr. Lily Edwards-Callaway, MS, PhD Assistant Professor Department of Animal Sciences Colorado State University   Dr. Noa Roman-Muniz, MS, DVM Professor and Director of Undergraduate Programs Department of Animal Sciences Colorado State University     If you have questions about your rights or welfare as a participant, please contact the CSU Institutional Review Board, at (970) 491-1553 or by email at RICRO_IRB@mail.colostate.edu.

Do you consent to participate in this research survey?

- Yes (1)
- No (3)

Skip To: End of Survey If Do you consent to participate in this research survey?  = No

End of Block: Introduction

Start of Block: Demographics

1. Do you currently work with preweaned dairy calves as a veterinarian?

- Yes (1)
- No (2)

Skip To: End of Survey If 1. Do you currently work with preweaned dairy calves as a veterinarian? = No

2. How old are you?

- <25 (1)
- 25-34 (2)
- 35-44 (3)
- 45-54 (4)
- 55-64 (5)
- 65 and older (6)
- Decline to answer (7)

3. What gender do you identify with?

- Man (1)
- Woman (2)
- Non-binary (3)
- Other (4)
- Decline to answer (5)

4. How long have you practiced veterinary medicine?

- Less than 1 year (1)
- 1-5 years (2)
- 6-10 years (3)
- 11-15 years (4)
- 16-20 (5)
- More than 20 years (6)
- Decline to answer (7)

5. In what region of the United States do you primarily work as a veterinarian?

- Midwest (ex. Illinois, Ohio, Wisconsin, Iowa, Kansas, Missouri) (1)
- Northeast (ex. Connecticut, Rhode Island, New Jersey, Pennsylvania) (4)
- South (ex. Florida, Maryland, Virginia, Alabama, Tennessee, Arkansas, Texas) (5)
- West (ex. Colorado, Montana, New Mexico, California, Washington) (6)
- Puerto Rico or other US territories (7)
- Outside of the United States (9)
- Other, specify: (8) __________________________________________________

6. Besides dairy cattle, which other animals do you currently work with as a veterinarian? Select all that apply.

- Beef cattle (1)
- Companion animals (Cats/dogs) (2)
- Horses (3)
- Poultry (4)
- Swine (5)
- None (6)
- Other, specify: (7) __________________________________________________

7. On average, how many separate facilities with preweaned dairy calves do you work with as the primary veterinarian?

▼ <5 (6) ... >20 (9)

8. On average, over the course of 12 months, what is the number of preweaned dairy calves on the production facilities that that you work?

▼ <50 (1) ... >5000 (7)

9. Do you work with any farms that are considered organic or do not allow the use of antibiotics?

- Yes (1)
- No (2)

End of Block: Demographics

Start of Block: Pain Perception Towards BRD and Fever

10. How painful do you consider BRD (pneumonia) to be for preweaned calves?

- no pain (1)
- mild (2)
- moderate (3)
- severe (4)
- very severe (5)
- worst pain imaginable (6)

11. Please explain your answer regarding how painful you consider BRD to be for preweaned dairy calves:

________________________________________________________________

________________________________________________________________

________________________________________________________________

________________________________________________________________

________________________________________________________________

12. How painful would you consider fever to be for preweaned calves?

- no pain (1)
- mild (2)
- moderate (3)
- severe (4)
- very severe (5)
- worst pain imaginable (6)

13. Please explain your answer regarding how painful you consider fever to be for preweaned dairy calves:

________________________________________________________________

________________________________________________________________

________________________________________________________________

________________________________________________________________

________________________________________________________________

14. Do you assess pain in preweaned calves with BRD in order to make treatment decisions?

- Yes (1)
- No (2)

Skip To: End of Block If 14. Do you assess pain in preweaned calves with BRD in order to make treatment decisions? = No

15. How important (from not at all important to extremely important) are each of the following when assessing pain associated with BRD in preweaned dairy calves? (adapted from de Oliveira et al., 2014; Huxley and Whay, 2006)

|  | Not at all important (1) | Slightly important (2) | Moderately important (3) | Very important (4) | Extremely important (5) |
| --- | --- | --- | --- | --- | --- |
| Appetite (16) |  |  |  |  |  |
| Attention towards the painful area (thorax) (17) |  |  |  |  |  |
| Back position (18) |  |  |  |  |  |
| Change in locomotion (19) |  |  |  |  |  |
| Change in posture (crouching, arched back, low head position) (20) |  |  |  |  |  |
| Ear position (21) |  |  |  |  |  |
| Facial expression (22) |  |  |  |  |  |
| Head position (23) |  |  |  |  |  |
| Interactive behavior (attention to tactile, visual, and/or auditory stimuli) (24) |  |  |  |  |  |
| Kicking/foot stomping (25) |  |  |  |  |  |
| Modification of social behavior (26) |  |  |  |  |  |
| Response to approach from human (27) |  |  |  |  |  |
| Teeth grinding (bruxism) (28) |  |  |  |  |  |
| Vocalization (29) |  |  |  |  |  |
| Other, please specify: (15) |  |  |  |  |  |

16. How important is the animal’s current pain-level when making a treatment decision regarding BRD for preweaned calves?

- Not at all important (5)
- Slightly important (4)
- Moderately important (3)
- Very important (2)
- Extremely important (1)

17. Do you consider your knowledge of recognizing and treating pain in preweaned dairy calves to be adequate?

- Yes (1)
- No (2)
- Other, please specify: (7) __________________________________________________

End of Block: Pain Perception Towards BRD and Fever

Start of Block: BRD Detection

18. How important (from not at all important to extremely important) are each of the following diagnostic tools in reaching a diagnosis of BRD in preweaned calves?

|  | Not at all important (1) | Slightly important (2) | Moderately important (3) | Very important (4) | Extremely important (5) |
| --- | --- | --- | --- | --- | --- |
| Auscultation (1) |  |  |  |  |  |
| Automated calf feeder alarms or data (9) |  |  |  |  |  |
| Behavioral observation (2) |  |  |  |  |  |
| Bronchoalveolar lavage (BAL) (10) |  |  |  |  |  |
| California Respiratory Scoring System (3) |  |  |  |  |  |
| Depression Score (4) |  |  |  |  |  |
| Feed intake (5) |  |  |  |  |  |
| Laboratory diagnostic testing including PCR, bacterial culture and sensitivity testing or virus isolation (11) |  |  |  |  |  |
| Nasopharyngeal or deep nasopharyngeal swabs (12) |  |  |  |  |  |
| On-farm necropsy of deceased animals (13) |  |  |  |  |  |
| Rectal Thermometer (14) |  |  |  |  |  |
| Remote Monitoring Systems (e.g. Allflex tags ®, Fevertags ®, Accelerometers) (15) |  |  |  |  |  |
| Thermography camera (16) |  |  |  |  |  |
| Ultrasound (6) |  |  |  |  |  |
| Whisper® Veterinary Stethoscope (17) |  |  |  |  |  |
| Wisconsin Respiratory Scoring System (7) |  |  |  |  |  |
| Other, specify: (8) |  |  |  |  |  |

19. How important (from not at all important to extremely important) are each of the following clinical signs in reaching a diagnosis of BRD in preweaned calves?

|  | Not at all important (1) | Slightly important (2) | Moderately important (3) | Very important (4) | Extremely important (5) |
| --- | --- | --- | --- | --- | --- |
| Abdominal breathing (1) |  |  |  |  |  |
| Clear (serous) nasal discharge (2) |  |  |  |  |  |
| Cloudy (purulent) nasal discharge (3) |  |  |  |  |  |
| Coughing (4) |  |  |  |  |  |
| Droopy ears (5) |  |  |  |  |  |
| Fever (6) |  |  |  |  |  |
| Lack of appetite (7) |  |  |  |  |  |
| Lethargy (8) |  |  |  |  |  |
| Inability to remain in sternal recumbency (9) |  |  |  |  |  |
| Inability to stand (10) |  |  |  |  |  |
| Increased respiratory effort (11) |  |  |  |  |  |
| Poor body condition (12) |  |  |  |  |  |
| Other, specify: (13) |  |  |  |  |  |

End of Block: BRD Detection

Start of Block: BRD Treatment

20. On average, how many antimicrobial treatments do you administer to a typical case of BRD in preweaned dairy calves that meet your case definition, before clinical signs resolve?

- 1 treatment (1)
- 2 treatments (4)
- 3 treatments (5)
- 4 treatments (6)
- 5 treatments (7)
- >5 treatments (8)

21. In the presence of continuing clinical signs that meet your case definition for BRD (e.g. fever, anorexia), how long do you typically wait before determining that the previous antimicrobial treatment was unsuccessful, and that retreatment with another antimicrobial is indicated?

- 12 to 24 hours (1)
- 24 to 48 hours (4)
- 48 to 96 hours (5)
- 4 -7 days (6)
- 7-14 days (7)
- >14 days (8)

End of Block: BRD Treatment

Start of Block: Question 22

| 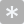 |
| --- |

22. Please rank your top 3 (type 1 for your first choice, 2 for your second choice and 3 for your third choice) antimicrobials that you, as a veterinarian, would select for **first line treatment** of BRD in preweaned dairy calves that meet your case definition.

______ Ceftiofur Crystalline Free Acid (e.g. Excede® 200mg) (11)

______ Danofloxacin Mesylate (e.g. Advocin® 180mg) (12)

______ Enrofloxacin (e.g. Baytril® 100mg) (13)

______ Florfenicol (e.g. Nuflor® 300mg) (14)

______ Gamithromycin (e.g. Zactran® 150mg) (15)

______ Oxytetracycline (e.g. Terramycin ® 250mg) (21)

______ Sulphonamides (Sustain III® ) (22)

______ Tildipirosin (e.g. Zuprevo® 180mg) (16)

______ Tilmicosin (e.g. Micotil® 300mg) (17)

______ Tulathromycin (e.g. Draxxin® 100mg) (18)

______ Other, specify: (19)

______ I would not select antibiotics or antimicrobials as my first line treatment for a preweaned calf with BRD. Why: (23)

End of Block: Question 22

Start of Block: Question 23

| 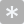 |
| --- |

23. Please rank your top three (type 1 for your first choice, 2 for your second choice and 3 for your third choice) antimicrobials that you, as a veterinarian, would select for **second line treatment** of BRD in preweaned dairy calves that meet your case definition.

______ Ceftiofur Crystalline Free Acid (e.g. Excede® 200mg) (11)

______ Danofloxacin Mesylate (e.g. Advocin® 180mg) (12)

______ Enrofloxacin (e.g. Baytril® 100mg) (13)

______ Florfenicol (e.g. Nuflor® 300mg) (14)

______ Gamithromycin (e.g. Zactran® 150mg) (15)

______ Oxytetracycline (e.g. Terramycin ® 250mg) (21)

______ Sulphonamides (Sustain III® ) (22)

______ Tildipirosin (e.g. Zuprevo® 180mg) (16)

______ Tilmicosin (e.g. Micotil® 300mg) (17)

______ Tulathromycin (e.g. Draxxin® 100mg) (18)

______ Other, specify: (19)

______ I would not select antibiotics or antimicrobials as my second line treatment for a preweaned calf with BRD. Why: (20)

End of Block: Question 23

Start of Block: Question 24

| 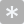 |
| --- |

24. Please rank your top three (type 1 for your first choice, 2 for your second choice and 3 for your third choice) antimicrobials that you would use to **control/ prevent BRD (provide metaphylaxis)** in preweaned dairy calves.

______ Ceftiofur Crystalline Free Acid (e.g. Excede® 200mg) (11)

______ Danofloxacin Mesylate (e.g. Advocin® 180mg) (12)

______ Enrofloxacin (e.g. Baytril® 100mg) (13)

______ Florfenicol (e.g. Nuflor® 300mg) (14)

______ Gamithromycin (e.g. Zactran® 150mg) (15)

______ Oxytetracycline (e.g. Terramycin ® 250mg) (21)

______ Sulphonamides (Sustain III® ) (22)

______ Tildipirosin (e.g. Zuprevo® 180mg) (16)

______ Tilmicosin (e.g. Micotil® 300mg) (17)

______ Tulathromycin (e.g. Draxxin® 100mg) (18)

______ Other, specify: (19)

______ I would not select antibiotics or antimicrobials to control/prevent BRD in a preweaned calf. Why: (20)

End of Block: Question 24

Start of Block: Q22 Additional Question

25. Please indicate the importance (from not at all important to extremely important) of the following reasons to use the drugs selected for your **first line of treatment**, in terms of their importance to treating BRD.

|  | Not at all important (1) | Slightly important (2) | Moderately important (3) | Very important (4) | Extremely important (5) |
| --- | --- | --- | --- | --- | --- |
| Antimicrobial Sensitivity of farm-specific pathogens (11) |  |  |  |  |  |
| Bactericidal (5) |  |  |  |  |  |
| Bacteriostatic (6) |  |  |  |  |  |
| Cost (7) |  |  |  |  |  |
| Decision or preference of the farm (9) |  |  |  |  |  |
| Drug Purchasing incentives/ Rebates (3) |  |  |  |  |  |
| Duration of activity after a single dose (14) |  |  |  |  |  |
| Easiest to administer (10) |  |  |  |  |  |
| Established Practice protocols (4) |  |  |  |  |  |
| Improves calf growth and/or feed intake (12) |  |  |  |  |  |
| Personal experience demonstrating effectiveness at resolving BRD (1) |  |  |  |  |  |
| Short meat withhold period (8) |  |  |  |  |  |
| Other, specify: (13) |  |  |  |  |  |

End of Block: Q22 Additional Question

Start of Block: BRD Treatment

26. Do you use antimicrobials in an extralabel manner to treat BRD?

- Yes (1)
- No (2)

Display This Question:

If 26. Do you use antimicrobials in an extralabel manner to treat BRD? = Yes

27. How often do you recommend the use of an antimicrobial in an extralabel manner under AMDUCA to treat BRD in preweaned calves?

- Never (1)
- Sometimes (4)
- About half of the time (5)
- Most of the time (6)
- Always (7)

End of Block: BRD Treatment

Start of Block: Ancillary Therapy

28. Given the choice between an antibiotic that contains an NSAID, an antibiotic alone, or an NSAID alone, which treatment option would you select for an animal that meets your case definition for BRD?

- Antibiotic only (1)
- Antibiotic with NSAID (4)
- NSAID Alone (5)

29. How frequently do you use drugs (other than antimicrobials) to reduce fever in a preweaned calf with BRD?

- Never (7)
- Sometimes (6)
- About half of the time (5)
- Most of the time (4)
- Always (1)

End of Block: Ancillary Therapy

Start of Block: Question 30

| 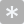 |
| --- |

30. Please rank your top 3 (type 1 for your first choice, 2 for your second choice and 3 for your third choice) non-steroidal anti-inflammatory drugs that you, as a veterinarian, would select when treating BRD in preweaned dairy calves that meet your case definition?

______ Aspirin (1)

______ Flunixin (e.g. Banamine®) Injection (2)

______ Flunixin (e.g. Banamine®) pour-on (3)

______ Ketoprofen (Anafen® Injection) (4)

______ Lidocaine (5)

______ Meloxicam Injection (Metacam® Injection) (6)

______ Meloxicam (Oral) (7)

______ Phenylbutazone (8)

______ Other, specify: (9)

______ I would not select non-steroidal anti-inflammatory drugs to treat a preweaned calf with BRD. Why: (10)

31. How do you decide if analgesics are warranted for a preweaned calf with BRD?

________________________________________________________________

________________________________________________________________

________________________________________________________________

________________________________________________________________

________________________________________________________________

End of Block: Question 30

Start of Block: Ancillary Therapy 2

32. Besides antibiotics, how important (from not at all important to extremely important) do you consider each of the following ancillary therapies to include in your treatment regimen for BRD?

|  | Not at all important (1) | Slightly important (2) | Moderately important (6) | Very important (7) | Extremely important (8) |
| --- | --- | --- | --- | --- | --- |
| Antihistamine (4) |  |  |  |  |  |
| Corticosteroids (e.g. dexamethasone) (2) |  |  |  |  |  |
| IV fluids/electrolytes (5) |  |  |  |  |  |
| Move the calf to a sick/hospital pen (6) |  |  |  |  |  |
| Non-steroidal anti-inflammatory Drugs (NSAIDs) (1) |  |  |  |  |  |
| Oral fluids/electrolytes via bottle (7) |  |  |  |  |  |
| Organic options such as garlic, essential oils, etc. (8) |  |  |  |  |  |
| Probiotics (9) |  |  |  |  |  |
| Revaccination with a BRD vaccine (10) |  |  |  |  |  |
| Vitamin C (11) |  |  |  |  |  |
| Other, specify: (12) |  |  |  |  |  |

33. How important (from not at all important to extremely important) are each of the following reasons when choosing to administer ancillary therapy to a preweaned calves with BRD.

|  | Not at all important (1) | Slightly important (2) | Moderately important (3) | Very important (4) | Extremely important (5) |
| --- | --- | --- | --- | --- | --- |
| Cost (1) |  |  |  |  |  |
| Decision or preference of the farm (2) |  |  |  |  |  |
| Easiest to administer (3) |  |  |  |  |  |
| Improves calf growth and/or feed intake (4) |  |  |  |  |  |
| Most effective at resolving BRD (5) |  |  |  |  |  |
| Most effective at reducing fever (6) |  |  |  |  |  |
| Most effective at relieving pain (7) |  |  |  |  |  |
| Most effective at improving animal welfare (9) |  |  |  |  |  |
| Other, specify: (8) |  |  |  |  |  |

End of Block: Ancillary Therapy 2

Start of Block: BRD Control

34. How important (from not at all important to extremely important) are each of the following control measures implemented to reduce the incidence of BRD in preweaned dairy calves on your production systems.

|  | Not at all important (1) | Slightly important (2) | Moderately important (3) | Very important (4) | Extremely important (5) |
| --- | --- | --- | --- | --- | --- |
| Autogenous vaccines (1) |  |  |  |  |  |
| Bedding management (2) |  |  |  |  |  |
| Choice of bedding material (3) |  |  |  |  |  |
| Colostrum management (4) |  |  |  |  |  |
| Group Housing (5) |  |  |  |  |  |
| Individual Housing (11) |  |  |  |  |  |
| Injectable vaccines against BRD pathogens (6) |  |  |  |  |  |
| Intranasal vaccine against BRD pathogens (7) |  |  |  |  |  |
| Mass medication with an injectable antimicrobial (metaphylaxis) (8) |  |  |  |  |  |
| Medicated feed (9) |  |  |  |  |  |
| Medicated Milk Replacer (10) |  |  |  |  |  |
| Medicated Water (15) |  |  |  |  |  |
| Probiotics (17) |  |  |  |  |  |
| Ventilation and facility improvements (12) |  |  |  |  |  |
| Vitamin supplementation (13) |  |  |  |  |  |
| Waste Milk Management (e.g. pasteurization) (14) |  |  |  |  |  |
| Other, specify: (16) |  |  |  |  |  |

35. What do you think future studies regarding calf health need to address?

________________________________________________________________

________________________________________________________________

________________________________________________________________

________________________________________________________________

________________________________________________________________

End of Block: BRD Control

Start of Block: Conclusion

Thank you for taking this survey. If you have any questions about this research project, please contact: Dr. Catie Cramer, MS, PhD (Principal Investigator)Assistant ProfessorDepartment of Animal SciencesColorado State Universitycatie.cramer@colostate.edu. Dr. Tanya Applegate, MS, DVM, DACVIM (LAIM)InstructorEquine Medicine and Livestock Veterinary ServicesColorado State University Dr. Hans Coetzee, BVSc, Cert CHP, PhD, DACVCP, DACAW, DECAWBMProfessor and HeadDepartment of Anatomy & PhysiologyKansas State University Dr. Lily Edwards-Callaway, MS, PhDAssistant ProfessorDepartment of Animal SciencesColorado State University Dr. Noa Roman-Muniz, MS, DVMProfessor and Director of Undergraduate ProgramsDepartment of Animal SciencesColorado State University  If you have questions about your rights or welfare as a participant, please contact the CSU Institutional Review Board, at (970) 491-1553 or by email at RICRO_IRB@mail.colostate.edu.

End of Block: Conclusion
